# Therapeutic advances in Marburg virus disease: from experimental treatments to vaccine development

**DOI:** 10.1097/MS9.0000000000003213

**Published:** 2025-03-28

**Authors:** Faida Paison, Pascal Ubuzima, Eugene Nshimiyimana, Janvier Habumugisha, Secret Atukunda, Fortunate Ayebare, Gustave Munyurangabo, Betty Amikoro, Biyun Su

**Affiliations:** a School of Education, Kigali Independent University ULK, Kigali, Rwanda; b Department of Preventive and Community Dentistry, School of Dentistry, College of Medicine and Health Sciences, University of Rwanda, Kigali, Rwanda; c Department of Orthodontics, Affiliated Hospital of Stomatology, Anhui Medical University, Hefei, Anhui, China; d Department of Biochemistry and Molecular Dentistry, Graduate School of Medicine, Dentistry and Pharmaceutical Sciences, Okayama University, Okayama, Japan; e Department of Orthodontics, Graduate School of Medicine, Dentistry and Pharmaceutical Sciences, Okayama University, Okayama, Japan; f School of Medicine, University of Global Health Equity, Butaro, Rwanda; g Department of Clinical Medicine and Community Health, School of Health Sciences, University of Rwanda, Kigali, Rwanda; h Department of Hematology, The Second Affiliated Hospital of Xi’an Jiaotong University, Xi’an, Shaanxi, People’s Republic of China; i Center for Tumor and Immunology, the Precision Medical Institute, Xi’an Jiaotong University, Xi’an, Shaanxi, People’s Republic of China; j Graduate School of Medicine and Surgery, Xi’an Jiaotong University, Xi’an, China; k College of Chemistry and Chemical Engineering, Xi’an Shiyou University, Xi’an, Shaanxi, China

**Keywords:** epidemic, Marburg virus (MARV), pathogenesis, prevention and control, treatment

## Abstract

The Marburg virus (MARV), discovered in 1967, has led to devastating outbreaks over the world; the mortality rate of Marburg virus disease (MVD) varies according to the outbreak and viral type. The very first known filovirus hemorrhagic fever outbreaks occurred in Germany and the former Yugoslavia. MVD is a deadly illness caused by the MARV virus, part of the Filoviridae family. It progresses with early viral replication that damages immune cells, followed by destruction of organs like the spleen, liver, and lymphoid tissues. Combatting this disease requires proper health education, and strong strategies. MVD is a lethal single-stranded RNA virus transmitted by *Egyptian rousette* bats, with a fatality rate of approximately 90%. This work explored ongoing studies on the recent vaccine developments and experimental therapies, such as a recombinant vesicular stomatitis virus (VSV)-based vaccine and MVA-BN-Filo, aiming to combat this deadly infection. Over the previous years, MARV has also spread to non-endemic African countries, demonstrating its potential to cause epidemics. Although MARV-specific vaccines are evaluated in preclinical and clinical research, none have been approved for human use. Studies revealed that Modified Vaccinia virus Ankara, a well-established viral vector used to generate vaccines against emerging pathogens, can deliver multiple antigens and has a remarkable clinical safety and immunogenicity record. MVD has been recently reported in Rwanda in 2024, an African country, and nearly 15 outbreaks of MVD have been reported. This review describes the nature of the MVD, key outbreaks, the virus’s pathogenesis, mode of transmission, clinical and laboratory diagnosis, and control and prevention measures to advance MVD treatment, drug development, vaccine creation, and prevention of MVD.

## Introduction

Marburg virus (MARV), a member of the Filoviridae family that also contains the Ebola virus (EBOV), causes Marburg virus disease (MVD), a zoonotic (animal-borne) virus whose reservoir is the Egyptian fruit bat (*Rousettus aegyptiacus*)^[1]^ as well as a very fatal disease with up to 88% case fatality rate^[2]^. MARV is a member of the filovirus family, causing severe Marburg disease. It is represented by two distinct viruses, MARV and Ravn virus (RAVV), with genomes of 19 000 nucleotides^[3]^.

**HIGHLIGHTS**

- Discovery and mortality variability of Marburg virus disease.
- Pathogenesis and disease progression of Marburg virus disease.
- Natural reservoirs and transmission of Marburg virus disease.
- Recent outbreaks of Marburg virus disease.
- Prevention and control strategies of Marburg virus disease.

MARV was discovered in August 1967, when laboratory workers in Marburg and Frankfurt, Germany, and Belgrade, Yugoslavia (now Serbia), were infected by a previously unknown infectious agent^[4]^, during laboratory research with infected grivet monkeys (*Chlorocebus aethiops*) brought from Uganda, Africa. Since then, MVD outbreaks have been documented across Africa, the most recent happening in Rwanda in 2024^[5]^. Previous reports of MVD outbreaks have come from African nations like the Democratic Republic of Congo (DRC), Angola, Kenya, Zimbabwe, Uganda, South Africa, and Guinea, Ghana as well as the United States, Netherlands, Yugoslavia, and Russia^[6]^. The most recent epidemic was recorded in Rwanda in the year 2024. Scientists isolated MARV from fruit bats in Uganda, indicating fruit bats as the viral reservoir. MVD outbreaks in central Africa suggest bats are infected, with African green monkeys and pigs as potential amplified hosts^[7]^.

The MARV can be transmitted to humans through infected bat feces or aerosols, as well as through contact with blood or body fluids from individuals with or who have succumbed to MVD^[8]^. The MARV can be transmitted through contaminated objects, semen, and contact with infected individuals. It can persist in the testicles and eye, but no evidence suggests it can be spread through sex or vaginal fluids from affected women^[9]^. MVD has an incubation period of 2–21 days and onsets suddenly, with symptoms including fever, chills, headache, myalgia, and maculopapular rash. Symptoms worsen and can cause jaundice, pancreatitis, weight loss, delirium, shock, liver failure, and hemorrhaging^[10]^.

The MARV can be prevented by avoiding contact with fruit bats and sick primates, using nursing techniques like Ebola, and implementing strict isolation for infected individuals^[11]^. The Ministries of Health mandated quarantine for newcomers from MVD outbreak countries, outlining guidelines for disease definition, precautions, and handling of suspected cases. Following the announcement of the virus epidemic by African states, Egypt also disclosed the sterilization and disinfection procedures it is implementing to prevent MARV sickness. A sodium hypochlorite solution is used for these disinfection procedures. To stop the MARV from entering the nation, Egypt is on high alert^[12]^. MVD diagnosis is challenging due to clinical similarities with other infectious or endemic diseases. Laboratory diagnosis includes enzyme-linked immunoassay (ELISA), polymerase chain reaction (PCR), immunoglobulin M (IgM) capture, and virus isolation^[13]^.

MARV infection treatment involves supportive care, including rest, hydration, oxygen, and specific symptoms treatment. Medications like acetaminophen relieve pain and fever, while intravenous fluids and blood transfusions stabilize electrolytes. Monoclonal antibody therapies and antiviral therapies like remdesivir and favipiravir may be used^[14]^. Supportive treatment for a patient with a blood clot includes balancing fluids, maintaining oxygen, replacing lost blood and clotting factors, and treating infections. Heparin treatment is controversial due to clotting factor consumption concerns. Hospital practices should include contact and droplet precautions^[15]^. Furthermore, certain medications that seem to be helpful for external ventricular drain (EVD) are probably not relevant for treating MVD because monoclonal antibodies (Mabs) are the most promising treatment for EVD. Similarly, there has been no evidence of cross-protection for MVD using immunization platforms such as the vesiculo stomatitis virus vaccine that uses antigens for EVD^[16]^. In light of this, thorough in-depth disease investigations and analysis may facilitate future medical research and help to improve the therapeutic management of MVD.

Existing knowledge about host-virus interactions that could facilitate designing and developing vaccines or therapeutics to control MVD seems to be shallow. An earlier study has identified GP and VP40 matrix proteins as the most potent antigenic viral protein candidates to develop chimeric subunit vaccines. Considering the need to bridge the knowledge gap about its pathogenicity, its potential aerosolized transmission, and the lack of immunological and pharmacological therapeutic measures, extensive research on MVD therapeutics is urgently required^[17]^. Therefore, this review explores potential candidate vaccines against MARV, epidemiology, pathogenesis, clinical manifestation, management, and the advances made in efforts to combat MVD.

## MARV: biology and pathogenesis

### Virus structure and genetics

Detailed description of the MARV, its morphology, genetic composition, and classification within the Filoviridae family. MARV is one of the lethal huma viruses with RAVV and is representatives of the species of Marburg Marburgvirus, which later renamed Orthomarburgvirus marburgense in 2023^[18]^, and they are representative of the single genus that is found in the genus Marburgvirus, which is also later renamed Orthomarburgvirus. Their family is called Filoviridae family which is now comprised of other separate genera of Orthomarburgvirus, Orthoebolavirus, Cuevavirus, Dianlovirus, Oblavirus, Striavirus, Tapjovirus, and Thamnovirus^[18,19]^. MARV’s genome structure is found to be single-stranded RNA (Ribonucleic acid), with negative polarity^[20]^.

The MARV virion, like other filoviruses, is enveloped and exhibits a filamentous shape, which can vary from long and twisted to U- or 6-shaped, and even branched forms^[21]^. This diversity in structure reflects the complexity of the virus. The virion contains ribonucleoprotein (RNP) complexes composed of the viral genome, which is a negative-sense single-stranded RNA, and several structural proteins^[22]^. These include the nucleoprotein (NP), the polymerase co-factor (VP35), the transcriptional activator (VP30), and the large protein (L), which are essential for replication and transcription of the viral genome^[23,24]^. Additionally, filovirid particles carry a matrix protein (VP40) that helps in virion assembly by forming a regular layer beneath the viral envelope, and a glycoprotein (GP1,2) complex, which forms surface spikes approximately 7 nm in diameter that facilitate viral entry into host cells^[25,26]^.

The genome of MARV, like other filoviruses, lacks a 5′-cap or 3′-poly(A) tail and spans about 13.1–20.9 kilobases in length^[27,28]^. The genome’s structure includes a leader and trailer sequence that plays a key role in replication and transcription, as they contain the promoters for these processes. The virus’s genome encodes 6–10 open reading frames (ORFs), with 5 of them being conserved across all filovirids and encoding core structural proteins such as NP, VP35, GP1,2, VP30, and L^[26,29]^. These ORFs are flanked by noncoding regions that contain the transcription initiation and termination sites, necessary for proper replication and transcription. In MARV, as in other filoviruses, replication involves the formation of antigenomic RNA, which serves as a template for the production of genomic RNA^[28]^. The entry of MARV into host cells is a complex, multistep process involving initial attachment, endocytosis, and fusion with the host cell membrane^[30]^. Upon encountering a host cell, the viral glycoprotein GP1,2, which is responsible for attachment and entry of target cells, binds to specific cellular receptors such as C-type lectins or TAM (Tyro3, Axl, and Mer) receptor protein kinases, facilitating viral attachment^[31,32]^. This interaction is essential for the virus to gain entry into the cell.

In some cases, therapeutic interventions are designed to block this binding and prevent viral infection^[32,33]^. After attachment, the virus is internalized through a process known as endocytosis. The MARV virion is engulfed into an endosomal vesicle, where it undergoes fusion with the host membrane^[25]^. This fusion is pH-dependent and occurs in the acidic environment of the late endosome. The viral glycoprotein GP2 is responsible for mediating this fusion event. Specifically, the N-terminal fusion loop of GP2 inserts into the endosomal membrane, disrupting it and allowing the release of the viral ribonucleoprotein (RNP) complex into the host cell’s cytoplasm, where it can initiate replication and transcription^[34,35]^.

### Pathogenesis

The pathogenesis of MARV is driven by its ability to replicate efficiently and evade the host immune system^[36]^. Once inside the host cell, the viral genome is transcribed and replicated in the cytoplasm. This process involves the viral RNA-dependent RNA polymerase (L) and the co-factor VP35, which are responsible for synthesizing mRNA and replicating the viral genome^[30]^. The virus produces several key proteins, including the nucleoprotein (NP) and glycoprotein (GP), which are essential for the assembly of new virions. The newly formed viral particles bud from the host cell, a process mediated by the matrix protein VP40, which plays a crucial role in the assembly of the viral envelope and the release of the virus from the host cell^[37]^.

**Figure 1.**
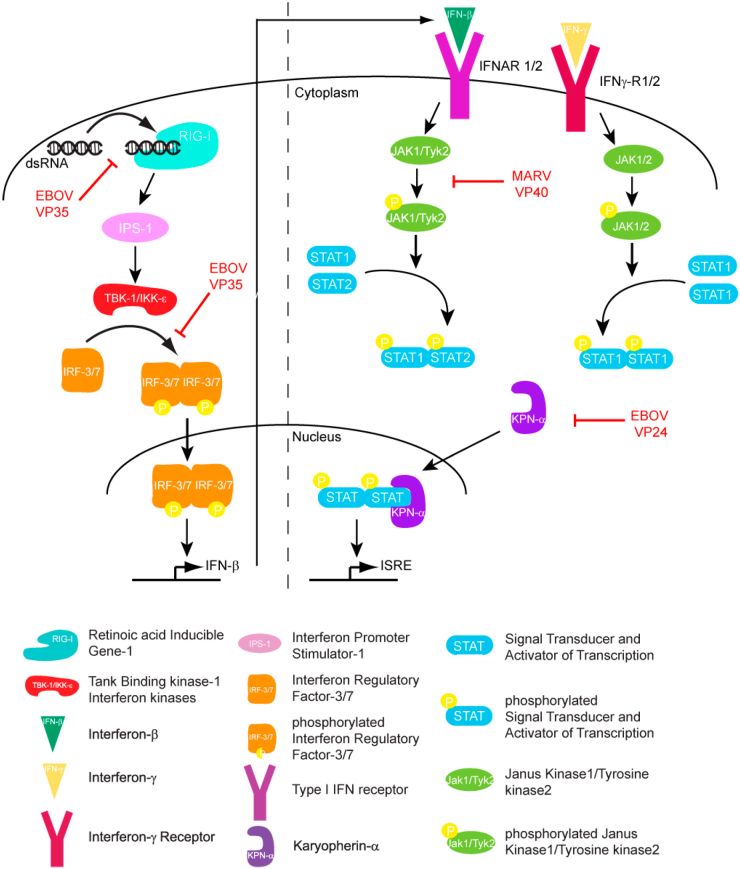
Filoviral proteins counter the host IFN response through multiple mechanisms in order to limit host antiviral responses, Figure and captions were taken from Ramanan et al. ^[43]^.

**Figure 2.**
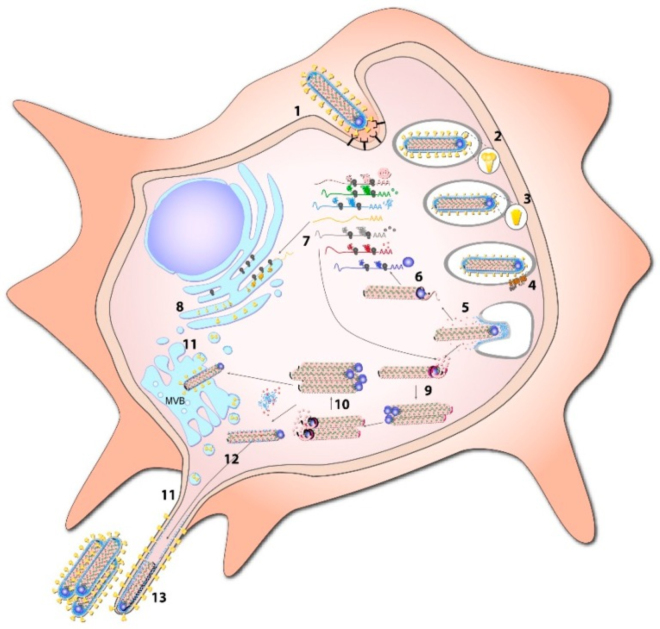
The replication cycle of MARV begins when the virus attaches to the surface of target cells by binding to attachment factors (1). Following endocytosis (2), the glycoprotein subunit GP1 is cleaved by endosomal proteases (3), facilitating binding to the Niemann-Pick C1 (NPC1) entry receptor (4). Fusion is then mediated in a pH-dependent manner by the glycoprotein subunit GP2. After the release of the viral nucleocapsid into the cytosol (5), transcription of the viral genome takes place (6), followed by mRNA translation by the host cell machinery (7). GP synthesis occurs in the endoplasmic reticulum (ER) and undergoes multiple post-translational modifications before being transported through the classical secretory pathway (8). Positive-sense antigenomes are synthesized from the incoming viral genomes (9), and these intermediate products serve as templates to replicate new negative-sense genomes (10). Following cleavage in the Golgi, GP is transported to multivesicular bodies (MVB) and to the cell membrane, where budding takes place, preferentially from filopodia (11). Nucleocapsids and VP24 are recruited to sites of viral budding (12), with the process primarily driven by VP40 (13). Figure and captions were taken from Schmidt and Mühlberger ^[44]^.

As MARV spreads through the body, it triggers a series of pathological changes. One of the hallmark features of infection is hemorrhagic manifestations, which include bleeding from the gums, internal bleeding, and disseminated intravascular coagulation (DIC). This is due to the destruction of endothelial cells and the disruption of blood vessel integrity^[38]^. The virus also induces a cytokine storm, a hyperactive immune response characterized by the excessive release of pro-inflammatory cytokines^[39,40]^. This leads to severe inflammation, tissue damage, and multi-organ failure, which are key contributors to the high mortality rate associated with the virus. MARV also employs several immune evasion mechanisms to bypass the host’s immune system. Like other filovirus, filoviral proteins help to inhibit the production of type I interferons, which are crucial for the innate immune response^[41]^, and immune strategies had been presented in Fig 1. This interference allows the virus to replicate unchecked during the early stages of infection. Additionally, MARV proteins disrupt antigen presentation, which impairs the adaptive immune system’s ability to recognize and target infected cells^[42,43]^. As a result, indicated in Fig 2, the virus can continue to replicate and spread throughout the body, overwhelming the host’s immune defenses^[4,44]^.

### Animal reservoirs and transmission dynamics

The natural reservoir for MARV is the Egyptian fruit bat (*R. aegyptiacus*), which is widely distributed across sub-Saharan Africa and parts of the Middle East^[7]^. These bats harbor the virus asymptomatically, playing a crucial role in the virus’s life cycle. Spillover events occur when humans come into contact with infected bats or their excretions, such as saliva, urine, or feces^[45,46]^. Additionally, nonhuman primates (NHPs) can serve as intermediate hosts, amplifying the virus before it spreads to humans. Human-to-human transmission occurs primarily through direct contact with the bodily fluids or tissues of infected individuals. This can happen during close contact with an infected person’s blood, vomit, feces, or other bodily fluids^[47]^. Nosocomial outbreaks, where the virus spreads in healthcare settings, have been a significant concern, highlighting the need for stringent infection control measures^[48,49]^. There is also evidence suggesting that aerosol transmission may occur under specific circumstances, although it is considered less common^[50]^.

### Immune response and viral evasion

The host immune response to viral infection including MARV involves both innate and adaptive components^[51]^. The innate immune system detects viral components through pattern recognition receptors, initiating the production of interferons and other antiviral cytokines^[52]^. However, MARV has evolved multiple strategies to counteract these defenses. For example, the viral VP35 protein inhibits the host’s ability to produce interferons, which are essential for the antiviral response. The innate immune system slows the viral infection to allow the time for antibody and T-Cell which come under the adaptive immune side^[51,53]^. Then, neutralizing antibodies plays a key role in controlling MARV infection. These antibodies target the viral glycoprotein GP, preventing the virus from entering host cells^[33,54]^. Cytotoxic T cells also contribute by killing infected cells and reducing viral replication^[55,56]^. However, MARV’s ability to suppress the immune response, combined with its rapid replication, often overwhelms the host immune system, contributing to the severity of the disease.

## Epidemiology of MVD

### Geographic distribution of MVD outbreaks

A total of ten MVD outbreaks have been documented, ranging from single cases to widespread community outbreaks involving hundreds of individuals. Animal-to-human transmission has been confirmed or suspected in five countries: Kenya, Uganda, Zimbabwe, Angola, and the DRC. In most instances, caves or mines were identified as the likely settings for spillover events^[57,58]^. Nevertheless, most documented animal infections were found in bats, typically investigated in response to human cases, except for one case involving grivets associated with the 1967 laboratory outbreak. These monkeys were captured near Kidera and Namsale in Uganda, where they were believed to have contracted the infection^[59]^. Furthermore, comprehensive knowledge of all human and animal MVD outbreaks is essential for understanding the risks associated with this disease. Previous studies suggest that the region at risk of zoonotic transmission extends far beyond the countries that have reported cases so far. Geological features appear to play a significant role in shaping areas of potential MARV risk. Most at-risk populations are concentrated in regions with prior outbreaks, particularly Uganda, Kenya, and the DRC. Notable at-risk countries that have yet to report human cases include Ethiopia, Cameroon, and Zambia, where large areas are predicted to be vulnerable^[7,60]^.

### Outbreaks of MVD

#### Marburg virus disease outbreak in Germany

MARV was first identified in August 1967 when laboratory workers in Marburg and Frankfurt, Germany, as well as Belgrade, Yugoslavia (present-day Serbia), were infected with an unknown pathogen. Among the 31 cases reported 25 primary and 6 secondary infections), the disease was severe and resulted in 7 fatalities^[4]^. Before the onset of the disease, all primary cases had either direct contact with grivets (*Chlorocebus aethiops*, Linnaeus, 1758), the infections were categorized into four groups. The first and largest group included individuals who had direct contact with the blood and organs of monkeys imported from Uganda. The second group comprised laboratory personnel who handled simian organs or cell culture materials. The third group consisted of individuals who contracted the infection through exposure to the blood of other patients^[61]^. The outbreak of the disease in Marburg, Frankfurt, and Belgrade was traced to a shared source: imported monkeys of the species *Cercopithecus aethiops* from Uganda, due to the near East crisis, these monkeys were transported to Germany and Yugoslavia via London, where they were temporarily housed at the airport alongside other animals. Seroepidemiological studies utilizing a complement-fixation antibody test suggested a high prevalence of positive reactions among monkeys from Uganda and other regions^[62]^. However, these findings were not corroborated by subsequent studies employing more specific methods. The reservoir and transmission mechanisms of the MARV remain unknown, This is supported by epizootiological and epidemiological studies, along with the current understanding of MARV endemicity in Africa and It is likely that the grivets were already infected with MARV before being exported from Uganda^[7,63]^.

The pathogen was named MARV, after the city with the highest number of cases, and it marked the first isolation of a filovirus. A study published in *The Lancet*, which incorrectly attributed the mysterious disease to rickettsia or chlamydia, has often been mistakenly cited as the initial report identifying the causative agent of MVD^[64]^. EBOV, the now more widely recognized member of the family, first emerged in Africa in 1976. Soon after, Marburg viruses and Ebolaviruses were grouped into a newly established family, Filoviridae, named for their characteristic thread-like morphology (with “filum” derived from the Latin word for thread)^[65]^.

#### MARV outbreak in South Africa

The 1975 MVD epidemic was the disease’s second known incidence and the first in Africa. It occurred in Johannesburg, South Africa, with three cases and one fatality. The outbreak began when a 20-year-old Australian man traveled to Zimbabwe (then Rhodesia) and visited numerous bat-infested caves. After returning to Johannesburg, he had symptoms such as fever, headache, myalgia, and vomiting before dying on 5 February 1975. His travel companion and the nurse who cared for him were secondary cases, and both survived with supportive care. Rapid patient isolation and contact tracking effectively restricted the outbreak. While the source of infection remained unknown, presumption was that the index patient acquired the virus from exposure to bats or their droppings within the caves^[66]^.

#### MARV outbreak in Kenya

The third known epidemic of MVD occurred in Kenya in 1980. The original patient caught the virus in western Kenya, which spread to a doctor in Nairobi who had intimate contact with the patient, resulting in severe hematemesis. However, no new cases of transmission were detected in medical settings. Surveillance investigations in western Kenya found no indication of Marburg-virus illness, but they did suggest the possibility of Ebola hemorrhagic fever (HF)^[67]^. In 1987, a single incidence of MVD epidemic surfaced in Kenya, centered on a 15-year-old Danish boy who had the virus and died as a result. The child had come into contact with the virus while exploring a cave inhabited by Egyptian fruit bats. This was the first reported occurrence of Ravn viral transmission, a close relative of the MARV responsible for MVD. Effective containment procedures were implemented, including patient isolation and contact tracking, which successfully prevented the spread of subsequent cases^[68]^.

#### MARV outbreak in DRC

In both 1998 and 2000, a single MVD epidemic occurred in Durba, Democratic Republic of the Congo. The infected individuals were gold miners who worked in a mine known to be home to Egyptian fruit bats, the virus’s natural host. This epidemic had 154 cases and regrettably resulted in 128 deaths, with an 83% case fatality rate. Surprisingly, this was the first example of a significant MVD epidemic and the first instance of a combination outbreak including MARV and Ravn virus. These two closely related viruses both contribute to MVD^[69]^.

#### MARV outbreak in Angola

The outbreak likely began in October 2004 in Uige Province, located in northern Angola, but it remained undiagnosed until March 2005. The outbreak involved 252 reported cases, with 227 fatalities (90%), according to a 2005 report by the Ministry of Health, Republic of Angola. This represents the largest recorded outbreak of Marburg HF to date^[70]^. The transmission of HF to healthcare workers during this period raised awareness within the community, prompting suspicion of Marburg or Ebola HF^[4]^.

#### Multidistrict outbreak of MVD in Uganda

Since 2000, Uganda has faced multiple outbreaks of viral hemorrhagic fevers (VHFs). EBOV outbreaks were reported in Gulu District in 2000, Bundibugyo in 2007, Luwero in 2011, Kibaale in July 2012, and again in Luwero in November 2012. MARV hemorrhagic fever (VHF) was first documented in Ibanda District in 2007. More recently, in 2012, two additional Marburg VHF outbreaks occurred in the districts of Ibanda and Kabale^[71]^. However, as of 13 November 2012, a total of 14 cases, comprising 9 confirmed and 5 probable cases, had been recorded, with 7 fatalities, resulting in a case fatality rate (CFR) of 50%. Among the 202 identified contacts, 193 had completed the 21-day follow-up period^[72]^.

#### MARV outbreak in Guinea

On 2 August 2021, a patient in the town of Temessadou M’Boké passed away, exhibiting hemorrhaging from various natural orifices. Postmortem analysis using real-time reverse transcription PCR on 3 August confirmed MARV as the cause. Field investigations were initiated, and the diagnosis was subsequently validated by two additional laboratories. Whole-genome sequencing using in-country metagenomic techniques recovered 99.3% of the MARV genome, with phylogenetic analysis revealing a relationship to strains found in bats from Sierra Leone and humans in Angola. A 21-day observation of all identified contacts revealed no symptoms, and no further cases emerged^[73]^.

Furthermore, Forest Guinea, along with other areas of West Africa including Sierra Leone, is predicted to be environmentally suitable for zoonotic transmission of MVD by bats and particularly by *R. aegyptiacus* which has been identified as a natural MARV reservoir host. Most MARV bat reservoir hosts are present in the forested region of Guinea, particularly in Koundou, which is close to the case emergence location. The patient had limited social interactions and lived in a household of four people. There was no evidence of travel history to country outside Guinea for the patient or his close contacts, nor contacts to returning travelers. He was a farmer living in close contact with nature and wildlife and may therefore have experienced repeated exposure to an environment or food contaminated with excreta of MARV-infected bats. Community surveys showed that while he may have harvested wild fruits for personal consumption. Both the epidemiological data and phylogenetic analysis strongly suggest that the newly identified MVD in Guinea is unlikely to have been imported. Instead, the evidence supports the hypothesis that its emergence resulted from a zoonotic transmission event originating from a bat reservoir in late July 2021^[74,75]^.

#### MARV outbreak in equatorial Guinea

February 2023, two villages in the Nsock Nsomo district of Kie-Ntem province experienced at least eight fatalities due to a virus outbreak. Health authorities collected blood samples from eight contacts, but real-time reverse transcriptase-PCR (RT-PCR) testing at Centre International de Recherches Medicales de Franceville yielded negative results. One sample was confirmed to be positive for the MARV, leading to a patient who died. As of 21 February, nine cases have been reported, all resulting in fatalities. Health workers have not been affected, and 34 contacts remain under surveillance. Concerns have been raised that the virus may be spreading undetected within the community^[76]^.

#### MARV outbreak in Rwanda

On 27 September 2024, Rwanda’s Ministry of Health announced the country’s first MVD outbreak, with healthcare workers in Kigali being notably impacted. As of 6 October, a total of 49 cases have been reported, including 12 fatalities, with healthcare workers in Kigali being disproportionately affected. Over 400 contacts were traced in order to curb further transmission and the World Health Organization (WHO) has designated this outbreak as a Grade 3 event, highlighting its complexity and emphasizing the necessity for a comprehensive and coordinated large-scale response^[77,78]^.

Furthermore, as of 24 October 2024, Rwanda has reported a total of 64 cases of MVD, including 15 fatalities, resulting in a CFR of 23.4%. Among the confirmed cases with available demographic data, 70% were male, and 48% were within the age group of 30–39 years^[79]^. Nevertheless, On 14 November 2024, Rwanda’s health minister declared the end of the MARV outbreak, as no new cases had been reported for nearly 2 weeks. The outbreak, first announced in late September, prompted the country to initiate vaccinations against the virus in October. While MARV typically has a fatality rate of up to 88%, Rwanda’s outbreak experienced a significantly lower mortality rate of approximately 23%, with 15 fatalities among 66 confirmed cases^[80]^.

### Risk factors

Healthcare occupations face the highest risk of exposure to MVD, with nursing staff being the most frequently affected (91%), followed by medical staff (81%) and laboratory staff (28%). Other exposed roles include medical auxiliaries, students, pharmacists, phlebotomists, radiographers, counselors, transporters, burial teams, a prisoner providing care to another prisoner, and a construction worker at a healthcare facility^[81]^. Furthermore, Individuals visiting caves are at high risk of exposure and should adopt protective measures, including wearing masks, gloves, personal protective equipment (PPE), or quarantining in suspected cases. Experimental studies have demonstrated that pigs are vulnerable to EBOV infections and can shed the virus, serving as amplifiers. Other animals should also be considered potential amplifiers unless proven otherwise. Pig farms require close monitoring to prevent infections from fruit bats, and during outbreaks, animal-derived products such as meat and blood must be properly cooked prior to consumption^[82]^. Table 1 represents MARV outbreak epidemiology trends in the incidence of cases, mortality, and CFR.

**Table 1:** Marburg virus outbreaks epidemiology trends in the incidence of cases, mortality, and case fatality rates

Month and year	Country	Number of cases	Deaths	CFR	Epidemiology	References
August 1967	Germany	24	5	20.83%	Infection from African green monkey’s infected tissues imported from Uganda	[83]
	Germany	6	2	33.33%		
	Yugoslavia	2	0	0.00%		
February 1975	South Africa	3	1	33.33%	Nearly uncertain, may be visiting to Chinhoyi caves	[84]
January 1980	Kenya: Nairobi	2	1	50.00%	Working near Kitum Cave, Mount Elgon National Park	[67]
August 1987	Kenya: Nairobi	1	1	100.00%	Visiting Kitum Cave, Mount Elgon National Park	[85]
1988	Russia	1	1	100.00%	Unexpected event in laboratory	[86]
1998–2000	DRC: Durba	154	128	83.12%	Infection developed during gold mining in Goroumbwa cave	[87]
2004–2005	Angola	252	227	90.08%	Uncertain	[47]
June 2007	Uganda: Kamwenge	4	1	25.00%	Infection developed during gold mining in Kitaka Cave	[88]
January 2008	USA	1	0	0.00%	Visiting a Python Cave in Maramagambo Forest	[89]
July 2008	The Netherlands	1	1	100.00%	Visiting a Python Cave in Maramagambo Forest	[7]
October 2012	Uganda: Kabale, Ibanda, and Kamwenge	15	4	26.67%	South‐western Uganda	[67]
October 2014	Uganda: Kampala	1	1	100.00%	Infection probably spread from Mengo Hospital	[90]
October 2017	Uganda: Kween	4	3	75.00%	Infections probably spread from a cave in Kaptum grazing ground or a cave on the slope of Mount Elgon	[91]
August 2021–September 2021	Guinea: Gueckedou	1	1	100.0%	Bat reservoir	[73]
2023	Tanzania	9	6	66.7%	Under investigation	[82]
2023	Equatorial Guinea	40	35	87.5%	Under investigation	[92]
September 2024	Rwanda: Kigali	66	15	23%	Bat revoir	[79,80]

### Ecological and anthropogenic factors influencing outbreaks

The Ebola outbreak in West Africa exposed significant weaknesses in global health systems and underscored the profound economic consequences of infectious disease outbreaks on developing nations. Furthermore, the emergence of recent infectious diseases, such as SARS, and MARV, has been associated with human activities many of which are also key contributors to biodiversity decline^[93]^. Although most emerging zoonotic viruses originate from wildlife, growing scientific evidence indicates that humans, rather than bats, are primarily responsible for triggering these spillover events. Additionally, human activities that modify the environment, such as intensive farming, wildlife trade, urbanization, and global travel, have increased the likelihood of viral spillover. These changes have contributed to the emergence of several major zoonotic viruses, some of which have led to global pandemics^[94]^.

#### Wildlife hunting and food contamination

Leroy analyzed the VHF outbreak in the DRC, tracing the initial case to a 55-year-old woman from a cluster of villages at the epidemic’s core. Their findings revealed that these villages had forest-based “twin” settlements, remnants of postindependence policies, where residents continued farming and hunting. These sites also attracted seasonal bat populations, including Ebola-carrying species and MVDs, which fed on palm nuts from an abandoned plantation. This interaction between humans and wildlife likely facilitated viral transmission^[95]^.

Hunters took advantage of the seasonal bat migration, frequently killing them with shotguns and selling the meat at village markets. Given the timing of the first death, Leroy *et al*^[95]^ proposed a transmission pathway: a man who purchased bat meat died from an unknown cause, infecting his daughter. The infection then spread to a 55-year-old woman who assisted in preparing the child’s body for burial. Furthermore, Studies have shown that fruit bats can shed the MARV through their saliva. This suggests that contamination of fruit or other foods in areas where bats are feeding or roosting might contribute to the virus’s transmission^[47]^.

#### International and intranational travels

In outbreaks that spread across multiple countries via human-to-human transmission, international travel is inherently a contributing factor. Similarly, within a single country, outbreaks that spread to multiple regions may have been facilitated by intranational travel, leading to secondary transmission between localities^[96]^. In one outbreak, the arrival of large numbers of international press members to affected areas hindered response efforts. International trade and travel contributed to 12 out of 45 (26.7%) filovirus outbreaks, while intranational trade and travel played a role in 15 (33.3%) of these outbreaks^[97]^.

#### Human population density and urbanization

Most filovirus outbreaks have occurred in remote areas, but urbanization likely played a significant role in the 2014–2016 Ebola regional epidemic. The outbreak, which began in Guinea in December 2013, reached the capital, Conakry, by 1 February 2014, and then spread to urban areas in Liberia, Sierra Leone, and Nigeria^[98]^. In most filovirus outbreaks, high population density areas were urban. However, in one instance in Uganda, a district with high population density was due to displaced individuals living in vulnerable conditions. Population density was identified as a factor in five filovirus outbreaks, and in four of these cases, the affected areas were urban^[99]^.

#### War and conflict

Armed conflicts contributed to outbreaks in two main ways: in some instances, previous wars had severely weakened the medical infrastructure of the affected area, while in others, ongoing conflicts made it difficult for health workers and responders to operate. War or armed conflict was directly cited as a contributing factor in five (11.1%) outbreaks^[100]^.

#### Behavior changes

Human behavior plays a crucial role in the spread of emerging infectious diseases. Understanding how communities behave during epidemics is vital for enhancing control measures^[101]^. Behavioral changes are often influenced by culture, which encompasses the shared beliefs, language, communication, practices, and social structures of various groups. Many infectious disease outbreaks spread due to these cultural factors. Since the emergence of HIV/AIDS, MARV, and Ebola, elements such as stigma, discrimination, misinformation, distrust, and lack of awareness have significantly contributed to their transmission^[102]^.

## Clinical features and diagnosis of MVD

### Clinical presentation

The MARV causes MVD, and its clinical presentations are mainly known based on the early signs and symptoms observed during its initial onset in 1967^[103]^. The disease incubation period varies between 2 and 21 days, with an average of 5–9 in humans^[104]^. Then after, the victims start experiencing a surge of the early symptoms that include but are not limited to high fever, chill, vomiting, headache, diarrhea, malaise, and pain in different parts of the body: myalgia, arthralgia, retro-orbital pain, loin pain, abdominal pain, among others^[105]^. Of note, at the onset of the epidemic, early detection might be challenging due to nonspecific characteristics of MVD that mirror common signs of other tropical diseases, notably malaria, rickettsia, typhoid, or yellow fever. Consequently, delayed diagnosis not only leads to an increase of unregistered patients who continue spreading disease in a community but also the victims are susceptible to a higher mortality rate than those who are diagnosed earlier and start receiving supportive care^[92,106,107]^.

In fact, the wider margin of MVD case fatality rate (23%–88%) is mainly attributed to factors such as access to healthcare, type of virulent strains, early detection, and supportive therapy^[106–108]^. Given that early detection is an imperative determinant of better prognosis, the healthcare provider should inquire about the patient’s travel history to see if the victim has been to the epidemic area or visited high-risk areas like caves that often serve as a home for *R. aegyptiacus*_MARV natural reservoir host^[109]^. Taken together, despite commonalities of MRD with other tropical febrile illness, these findings highlight the importance of early MRD identification to avert high mortality cases, and the spread of epidemic. Nevertheless, MARV manifestations basically involve three main phases. The initial stage, the generalization phase, lasts about 5 days following MVD onset. At the end of first phase, patients might also suffer from dysphasia, conjunctivitis, pharyngitis, and viral exanthema. The latter is also recognized as the hallmark of filovirus infection. Moreover, lymphadenopathy, leukopenia, thrombocytopenia, and anemia have been reported. The second stage is marked by early organ complications that start around 5–13 days following the symptoms’ manifestation. The central nervous system is probably the most susceptible organ, hence encephalitis, confusion, irritability, and aggressions^[108,110]^. Next, lymphatic and vascular systems lead to enhanced vascular permeability and edema^[111]^.

In fact, three-quarters of patients present with hemorrhage that occurs in a wide range of areas, from mucosal to the gastrointestinal tract to the skin, especially venipuncture sites and bleedings from the nose, gums, and vagina. Other organs include the pancreas, liver, and kidneys, thanks to their enrichment of reticuloendothelial cells that act as viral tropic cells. Hence, the elevated serum level of essential enzymes such as alanine aminotransferase (ALT), and aspartate aminotransferase (AST) are also key signatures of MRV infection. The convalescent/late organ phase is the last phase that begins around the 13th–20th day of infection. The fatality case usually occurs between the 8th–16th day of MRD as a result of dehydration, shock, and multi-organ failure^[108,110]^. The fatal case(s) exhibit exacerbated neurological symptoms, notably seizure, confusion, aggression, and comma. Indeed, various studies reported severe hepatitis, myalgia, arthralgia, peeling of the rash-affected skin, and secondary infections, as well as spontaneous miscarriages among pregnant women. In contrast, most of the symptoms mentioned above might not present among recovery patients, who usually start rehabilitation on the 20th day of infection and beyond. However, the patients may recover from illness once they receive decent supportive care, or the disease might progress into fatal illness due to late organ failure that culminates into severe dehydration, shock, and comma^[112]^. Nevertheless, MVD features resemble flu-like symptoms and viral exanthema at the beginning, followed by hemorrhagic fever and spontaneous bleeding, especially at venipuncture sites, and neurological symptoms.

### Differential diagnosis

Filoviruses biologically share similar core features that could serve as promising therapeutic targets. For instance, in one study, the authors revealed that MARV VP35 and EBOV nucleoprotein share a similar core binding site, which can be draggable^[113]^. In the other study, eritoran_toll-like receptor four could prevent cytokine storms commonly occurring during bacterial, MARV, Ebola, and influenza infection^[114]^. Moreover, MARV and EBOV utilize RAB11 to hijack VP40 particle formation and budding^[115]^. Indeed, another independent study demonstrated that except in Lassa viruses, the CCZ1 (guanine nucleotide exchange factor) imposes a broad inhibition effect on filoviruses, including Severe Acute Respiratory Syndrome Coronavirus 2 (SARS-CoV-2), MARV, and EBOV, mainly by abolishing replication ability to drive transit from early-to-late endosomal trafficking^[116]^. Infection with SARS-CoV-2 variants of concern, SARS-CoV, and MERS-CoV further validates the antiviral effectiveness of tubeimosides and NPC1 function. As a result, tubeimosides show potential as a novel defense against highly pathogenic human coronaviruses and filoviruses, for which NPC1 serves as a crucial entrance co-factor in the late endosomes^[117]^. These studies further fortify that targeting vesicle formation, packaging, and trafficking are plausible ways to prevent these viruses.

### Diagnostic methods

Similar biological features across the febrile viruses in particular filoviruses, underpin common symptoms and signs at the onset of disease and add a further layer of complexity to the design of reliable diagnostic tools. Nevertheless, a holistic diagnostic approach could avert inaccurate results. So far, a positive reverse transcriptase PCR (RT-PCR) for MARV from blood or an oral (buccal membrane) swab is the primary confirmatory test for MARV infection^[118]^. In addition to its relatively better sensitivity and specificity, RT-PCR significantly reduce turnaround time (usually less than 2 hours) compared to other diagnostic tests like ELISA that take 4–48 hours^[119]^. In clinical settings, serological assays help estimate the stage of infection, whereby MRV antigen tests based on IgM and IgG indicate early and late (6–18 years) infection, respectively^[120]^. Moreover, the activated partial thromboplastin time and/or prolonged prothrombin time coagulation studies could correlate the MRV with severe symptoms such as DIC and other bleeding impairment^[121]^. As indicators of organ damage, enzyme assays such as serum amylase, liver enzyme assays, notably ALT, AST, and measurement of creatinine and electrolyte levels are imperative to infer the severity of pancreatitis, hepatitis, and renal damage. Complementary to this, imaging and histopathological analysis reveal pathological abnormalities in the above-mentioned vital organs^[118,122]^. Importantly, blood culture enables clinicians to rule out a bacterial infection or enteric fever, leading to the utilization of more specific antibiotics that can hamper viral pathogenesis^[123]^.

## Treatment and management of MVD

### Current treatment options of MVD

MVD is a rare but severe hemorrhagic fever caused by the MARV, a member of the Filoviridae family, which includes the closely related EBOV. Due to the high mortality rate, often around 90% during outbreaks, effective management of MVD remains a critical public health challenge^[59]^. Currently, there are no specific antiviral therapies approved for the treatment of MVD, making supportive care the cornerstone of clinical management^[124]^. Supportive interventions are aimed at mitigating symptoms and preventing complications to improve survival rates. Key elements of supportive care include aggressive fluid resuscitation to counteract dehydration caused by vomiting, diarrhea, and high fever^[122]^. Maintaining electrolyte balance is crucial to avoid life-threatening imbalances, such as hypokalemia or metabolic acidosis, that often occur in severe cases. Organ support may also be necessary, particularly in patients with multi-organ dysfunction syndrome, which frequently develops in the later stages of MVD^[125]^.

Mechanical ventilation may be required for respiratory failure, while renal replacement therapy can be life-saving in cases of acute kidney injury. The management of secondary bacterial infections is an essential component of supportive care, as the immunosuppression caused by MARV infection predisposes patients to opportunistic infections. Broad-spectrum antibiotics are often used prophylactically or upon clinical suspicion of sepsis^[126]^. Hemorrhagic complications, including internal bleeding and coagulopathy, are hallmark features of MVD and require prompt intervention^[69]^. Transfusions of blood products, such as platelets, fresh frozen plasma, and clotting factors, are used to stabilize coagulation and prevent catastrophic bleeding events^[125]^. While supportive care improves outcomes, the absence of specific antiviral agents underscores the urgent need for effective targeted therapies. Experimental treatments, though not yet widely available, represent a significant step forward in the fight against this deadly disease. And knowledge and attitudes of the community should be enhanced in the publish health^[127]^.

### *Experimental treatments and vaccines of* MVD

mAbs and experimental antiviral drugs (e.g. favipiravir, remdesivir). Vaccine development efforts: progress, challenges, and candidate vaccines (e.g. adenovirus-vectored vaccines). Significant progress has been made in developing experimental treatments and vaccines for MVD, driven by the urgent need for effective countermeasures^[128]^. Among the most promising therapies under investigation are mAbs and antiviral drugs, which target various stages of the viral life cycle. For instance, the monoclonal antibody MR191-N has shown remarkable efficacy in NHP studies, conferring up to 100% survival when administered within 5 days postinfection^[129]^. Such findings highlight the potential of antibody-based therapies to neutralize the virus and prevent disease progression. Antiviral drugs, including favipiravir (T-705) and remdesivir (GS-5734), have also shown promise^[130,131]^. Favipiravir, originally developed for influenza, acts by inhibiting viral RNA polymerase, while remdesivir, an adenosine nucleoside analog, interferes with viral RNA synthesis^[132]^.

Preclinical studies have demonstrated their efficacy in animal models of MARV infection. For instance, oral administration of favipiravir in mice resulted in complete survival, while intravenous administration in NHPs achieved an 83% survival rate. Combination therapies, such as pairing mAbs with remdesivir, have further enhanced therapeutic outcomes, offering a synergistic approach to combating the virus^[133,134]^. Studies have demonstrated significant survival benefits when such combinations are used, even in cases of delayed treatment.

Vaccine development for MARV has also seen substantial progress^[135–137]^. An ideal vaccine should provide broad protection not only against the MARV but also against all eight members of the Filoviridae family, including potentially unencountered species^[138]^. This cross-protection is essential given the unpredictable nature of filovirus outbreaks. Prophylactic vaccination strategies are particularly important for at-risk populations, such as healthcare workers, laboratory personnel, and communities living near endemic regions. Reactive vaccination during outbreaks could also play a critical role in containing the spread of the virus^[136]^. Current candidate vaccines include viral vector-based platforms and DNA vaccines. Viral vector vaccines, such as those using adenoviruses or vesicular stomatitis viruses (VSV), deliver MARV glycoproteins (GP) to elicit strong immune responses^[139]^. These have shown protective efficacy in preclinical studies, including small animals and NHP models. According to different criteria, WHO technical advisory group-candidate vaccine prioritization in October 2024 recommended four vaccine to enter into next different phases, including VSV-vectored vaccines developed by Public Health Vaccines in the USA and the other by the International AIDS Vaccine Initiative, ChAd3-vectored vaccine developed by Sabin Institute Vaccine, ChAdOx1-vectored vaccine by the University of Oxford^[140]^.

Another innovative approach is the development of pan-filovirus T-cell vaccines, which aim to induce robust CD8+ T-cell responses by targeting conserved viral epitopes across filovirus species^[141,142]^. These platforms represent a significant advancement in vaccine technology and offer hope for broader and more durable immunity^[143]^. The development of pan-filovirus vaccines is crucial for better managing future outbreaks. Since EBOV and MARV often occur in the same regions, a multivalent vaccine could offer broad protection and reduce the impact of outbreaks. Recent advancements have shown that virus-like particles (VLPs), expressing filovirus glycoproteins (GP), can effectively protect against homologous challenges. Studies demonstrated that hybrid VLPs containing both EBOV and MARV antigens offered protection in guinea pigs, eliciting robust immune responses^[141]^. Additionally, multivalent vaccines combining EBOV and MARV or SUDV have shown promising results, with single-dose vaccination protecting against both viruses^[144]^. Another notable promising filovirus vaccine is thermostable filovirus against SUDV alone or both SUDV and MARV^[145]^. In addition, one dosage of attenuated rVSV-N4CT1 vectors, each of which expresses a distinct filovirus GP, may offer defense against the filoviruses including EBOV, SUDV, and MARV^[146]^. Vaccines like VSV-based and Ad26-based platforms, expressing multiple filovirus GPs, have also shown efficacy in preclinical trials^[147]^. These and other prospective studies undergoing indicate strong potential for broader filovirus protection. These developments pave the way for vaccines capable of offering rapid, broad-spectrum protection in endemic regions. Despite these advancements, vaccine development faces challenges, including ensuring safety, achieving long-term immunity, and addressing logistical hurdles in vaccine deployment. Despite these hurdles, multivalent vaccine platforms hold promise for addressing filovirus outbreaks, providing a critical tool for global health security. Continued research, funding, and international collaboration are essential to translate these promising candidates into widely available public health tools.

### Challenges in therapeutic advances in treatment and vaccines of MVD

Despite the progress in experimental therapies and vaccine development, several challenges impede the effective treatment and prevention of MVD. One of the most significant obstacles is delays in diagnosis and treatment which are common, contributing to high mortality rates and further transmission of the virus, this is due to the lack of access to the capable healthcare infrastructure in outbreak-prone regions, typically located in remote and resource-limited areas of sub-Saharan Africa^[148]^. The cost and limited availability of experimental drugs and vaccines present additional barriers^[149]^. Many of the promising mAbs and antiviral drugs are still in preclinical or early clinical trial phases and have not been scaled for widespread use. Even when these treatments become available, their high production costs may render them inaccessible to the populations most in need. The complex biotechnological processes required for large-scale manufacturing, coupled with stringent regulatory approvals, further drive-up costs, which may limit access in low-resource settings. The logistical challenges of distributing these therapies to remote or outbreak-prone regions, where healthcare infrastructure may be limited, complicate timely access to life-saving treatments.

Similarly, vaccine deployment faces logistical challenges, particularly in reaching remote communities and maintaining cold-chain requirements for storage and transport. Additionally, vaccine manufacturing costs and limited production capacity can hinder large-scale availability, while public trust and vaccine hesitancy in affected regions may further complicate immunization efforts.

Underreporting of cases and delays in identifying outbreaks exacerbate these issues. Many cases of MVD may go undiagnosed due to limited laboratory capabilities and the nonspecific early symptoms of the disease, which can mimic other febrile illnesses such as malaria or typhoid fever^[150]^. This underreporting hampers timely responses and complicates efforts to evaluate the efficacy of experimental treatments during outbreaks.

Additionally, the unpredictable nature of MARV outbreaks complicates the design and implementation of clinical trials due to its viral genetic variability^[151]^. The sporadic occurrence of cases makes it difficult to enroll sufficient participants for robust studies, slowing the progress of drug and vaccine development^[152]^. Ethical considerations in testing experimental treatments during outbreaks also pose challenges, particularly in balancing the urgency of saving lives with the need for rigorous scientific evaluation. To overcome these barriers, global collaboration is essential. Strengthening healthcare systems in outbreak-prone regions, improving diagnostic capabilities, and investing in the development and equitable distribution of treatments and vaccines are critical steps^[153]^. Integrating MVD preparedness into broader global health strategies will ensure a more effective response to future outbreaks and reduce the devastating impact of this lethal disease.

## Control measures and response strategies

### Prevention and containment

To prevent MARV outbreaks, proactive measures at personal and national levels are crucial. Strong surveillance and response capabilities are essential for identifying and containing outbreaks. As COVID-19 progresses, alarming monkeypox cases and SARS-CoV-2 variants emerge^[154,155]^. However, effective preparedness and community engagement are vital for reducing the public health impact of MARV outbreaks. Key strategies include strengthening surveillance systems for early outbreak detection, improving laboratory capacity for rapid diagnosis, developing emergency response plans, training healthcare workers in prevention and control, and advancing research on vaccines and treatments. Public awareness campaigns and community involvement are equally important to enhance understanding of the virus, encourage prompt healthcare-seeking behavior, and support outbreak response efforts^[156,157]^ Additionally, patients should be isolated in treatment units and transferred to appropriate facilities to prevent infection spread. Healthcare workers should adhere to infection prevention protocols, wearing PPE, and ensure proper communication with the receiving facility for safe patient handover. Home management increases transmission risk^[158]^.

### Public health and community interventions

The WHO emphasizes that effective outbreak control involves several measures, namely minimizing bat-to-human transmission from exposure to fruit bat habitats, reducing human-to-human transmission through contact with infected patients or their body fluids, and addressing the risk of sexual transmission. Male survivors of MVD are advised to practice safe sex and hygiene for 12 months after symptoms appear or until two consecutive tests confirm their semen is free of the virus^[159]^.

Previous outbreaks have shown far lower death rates as a result of the successful treatment strategies. In addition, the timely action had been effective in reducing the illness^[61]^. Furthermore, given the similarity of MVD symptoms to those of typhoid, malaria, and other viral diseases, differential diagnostic testing is essential for all suspected cases. A global initiative is needed to ensure access to diagnostic facilities in affected regions and the authorities should promptly test and confirm suspected cases and manage MVD patients in dedicated hospital wards to prevent both primary infections and secondary transmission^[92]^.

MVD outbreaks have brought attention to the urgent need for evidence-based, customized, and flexible risk communication and community engagement (RCCE). RCCE practitioners must ensure supportive engagement with individuals performing burial rituals, promoting adherence to infection prevention and control measures while maintaining a culturally respectful and dignified process because it was found that funeral rituals involving close contact with the deceased, such as washing the body or handling personal belongings, shown significant increase in the risks of MVD transmission in outbreak areas^[156,160]^.

Emergency medical teams deployed as VHF response units require specialized skills and robust logistical and operational support to effectively care for patients with highly infectious diseases, such as MVD ^[161]^. Rapid response during sudden emergencies improves survival rates and reduces morbidity. Early deployment of medical teams for treatment and isolation is critical to building community trust and alleviating the strain on nonspecialized health facilities. While teams can self-determine their deployment timing, they are expected to demonstrate readiness to mobilize and become operational within 72 hours of a disaster, ensuring a coordinated response and effective referral systems. Larger teams, due to their complexity and role in secondary and tertiary care, must maintain even higher levels of preparedness^[162]^.

### International collaboration and challenges

Once there is an MVD outbreak, it can have worldwide consequences owing to increased travel, migration, and commerce, potentially causing outbreaks in nonendemic locations. As a result, including and inspecting MVD instances requires a multidisciplinary effort^[163,164]^. International collaboration, research funding, and capacity building are required for addressing the MVD epidemic. MVD should be prioritized for surveillance and preparedness under the global health security agenda, a global partnership aiming at preventing, detecting, and reacting to infectious disease threats. Public health groups, politicians, and researchers may all collaborate to successfully combat MVD by exchanging knowledge and resources^[165]^. MVD has been declared as a global public health emergency, thus it must receive the attention it deserves. To enhance MVD treatment options, it is critical to find novel pharmacological targets and conduct clinical studies to assess their safety and efficacy. To accelerate the discovery and approval of potential medicines, worldwide scientists, pharmaceutical firms, and regulatory agencies must collaborate^[166]^. A research by Brauburger *et al*^[4]^ found that MVD infection can lead to hair loss, testicular shrinkage, liver inflammation, encephalitis, skin desquamation, hyperhidrosis, forgetfulness, and reduced libido. Current clinical investigations are inadequate and there is no detailed knowledge about host-virus interaction for vaccine and therapeutic drug design. Enhancing awareness programs, collaborating with researchers, public health experts, policymakers, and biologists, and upgrading surveillance and monitoring are crucial. Circulation, wastewater monitoring, and prognosis of outbreaks are also important. Further analyses are needed to understand the relationship between MARV and the environment^[167]^. Therefore, by fostering a collaborative learning process, the world will be able to build on the successes and lessons learned from previous MVD response projects, adjusting its methods as needed^[168]^.

### Future challenges and outbreak management

The WHO highlights climate change and global warming as the most pressing global health concerns, with extensive evidence linking them to environmental disruption, vector insect exposure, and infectious disease propagation. The WHO works with international organizations, governments, and nongovernmental organizations to create comprehensive outbreak response plans. These partnerships facilitate quick deployment of resources, research, and logistical support to areas affected by the virus^[169]^. The interaction and epidemiological link between climate changes, environmental and ecological disruption, vector insect exposure, and infectious disease propagation has been extensively established. Understanding this relationship allows us to forecast the consequences of future climate changes on infectious agent ecology, disease propagation, vectors (intermediate hosts), reservoir animals, and ultimate hosts^[170]^. Human host factors play a prime role in emerging and reemerging viral diseases. As in other infectious diseases, people who are immunocompromised or have underlying medical conditions may be at increased risk for having poor outcomes from emerging viral diseases. MARV can be introduced to human populations through inhalation of the contaminated excreta from bats, with occasional secondary human-to-human transmission^[38]^. Challenges posed by the lack of knowledge on the pathogenicity and transmission dynamics of MARV. Emerging viral infections have affected the entire course of human history and have been the source of immeasurable human misery and death^[171]^.

Currently, there are no recognized or licensed antiviral therapies for MVD, therefore medical therapy consists mostly on supportive care. This involves rehydrating, controlling symptoms, and maintaining electrolytes balanced. In animal investigations, experimental therapies such as Galidesivir (BCX4430), an antiviral drug that works by terminating RNA chains and reducing the activity of viral RNA polymerase, resulted in higher survival rates and lower levels of viremia^[16,172]^. However, it is crucial to highlight that results from human trials on Galidesivir’s efficacy have yet to be published. Nonetheless, preliminary experimental trials showed that Galidesivir is safe and acceptable for healthy individuals, with no fatalities or serious adverse effects^[173]^. For the current outbreak in Rwanda, remdesivir is being offered experimentally as part of a post-exposure prophylaxis regimen, and is also available for separate treatment and in combination with Monoclonal Antibody Treatment (MBP091)^[112]^.

## Conclusion and future perspectives

The MARV, a highly virulent VHF, poses a significant global challenge due to its fatal outbreaks and clinical challenges. The lack of specific antiviral therapies or vaccines further emphasizes the need for comprehensive prevention and control strategies. Rapid, coordinated responses involving healthcare providers, researchers, and governmental bodies are crucial to curtail the impact of MARV outbreaks. The global perspective on MARV emphasizes the importance of preparedness, collaboration, and innovation in addressing this ongoing threat to public health.

Lessons learned from past outbreaks underscore the importance of preparedness, collaboration, and innovation in averting future outbreaks and minimizing their toll on human lives. Current management focuses on supportive care, while research focuses on developing specific antiviral treatments and vaccines. Early detection, rapid response, improved healthcare infrastructure, international collaboration, surveillance strengthening, and public health investment are vital for addressing MVD globally. However, understanding the MARV infection’s pathogenesis and asymptomatic development is crucial.

Clinical trials for treatment and vaccination are needed to gain FDA approval. Global efforts from epidemiologists, diagnosticians, researchers, and health agencies are needed to tackle the disease efficiently. Key research directions include elucidating MARV transmission mechanisms, developing effective preventative measures, and strengthening healthcare infrastructure in regions susceptible to outbreaks. Advances in vaccine technologies and small molecule antiviral compounds can help. Collaborative studies and research are essential for a comprehensive understanding of the virus’s life cycle.

## Data Availability

Data sharing does not apply to this article as no new data were created or analyzed in this study.
